# The T-box transcription factor TBX3 drives proliferation by direct repression of the p21^WAF1^ cyclin-dependent kinase inhibitor

**DOI:** 10.1186/s13008-016-0019-0

**Published:** 2016-04-22

**Authors:** Tarryn Willmer, Shannagh Hare, Jade Peres, Sharon Prince

**Affiliations:** Division of Cell Biology, Department of Human Biology, Faculty of Health Sciences, University of Cape Town, Observatory, Cape Town, 7925 South Africa

**Keywords:** TBX3, Transcription factor, p21^WAF1^, Cancer

## Abstract

**Background:**

TBX3, a member of the T-box family of transcription factors, is essential in development and has emerged as an important player in the oncogenic process. TBX3 is overexpressed in several cancers and has been shown to contribute directly to tumour formation, migration and invasion. However, little is known about the molecular basis for its role in development and oncogenesis because there is a paucity of information regarding its target genes. The cyclin-dependent kinase inhibitor p21^WAF1^ plays a pivotal role in a myriad of processes including cell cycle arrest, senescence and apoptosis and here we provide a detailed mechanism to show that it is a direct and biologically relevant target of TBX3.

**Results:**

Using a combination of luciferase reporter gene assays and in vitro and in vivo binding assays we show that TBX3 directly represses the p21^WAF1^ promoter by binding a T-element close to its initiator. Furthermore, we show that the TBX3 DNA binding domain is required for the transcriptional repression of p21^WAF1^ and that pseudo-phosphorylation of a serine proline motif (S190) located within this domain may play an important role in regulating this ability. Importantly, we demonstrate using knockdown and overexpression experiments that p21^WAF1^ repression by TBX3 is biologically significant and required for TBX3-induced cell proliferation of chondrosarcoma cells.

**Conclusions:**

Results from this study provide a detailed mechanism of how TBX3 transcriptionally represses p21^WAF1^ which adds to our understanding of how it may contribute to oncogenesis.

## Background

Members of the T-box transcription factor family play crucial roles in embryonic development [1–4] and there is overwhelming evidence to show that they impact on several cancers by functioning as tumour suppressors and/or tumour promoters [5–13]. For example, TBX3 is critical for the formation of, amongst other structures, the heart, limbs and mammary glands and haploinsufficiency of the human TBX3 gene results in ulnar-mammary syndrome which is characterized by hypoplasia of these structures [14–16]. On the other hand, the overexpression of TBX3 is a feature of a wide range of cancers and it is a key driver of several oncogenic processes including proliferation, migration and invasion [9, 11, 12, 16–20]. There are also a few studies that have described a tumour suppressor role for TBX3 in cervical, uterine and bladder tumours, glioblastomas and fibrosarcomas [13, 16]. Considering the plethora of biological processes that it contributes to, TBX3 must clearly be regulating a number of genes. However, to date very few TBX3 target genes have been identified.

An intact p19ARF-Mdm2-p53-p21^WAF^ pathway is important for protecting cells from becoming cancerous because it negatively regulates the cell cycle and promotes senescence and apoptosis [21]. The cyclin-dependent kinase inhibitor p14ARF is responsible for the inhibition of MDM2-mediated ubiquitination and subsequent degradation of p53 [22, 23]. TBX3 is able to bypass senescence and/or evade apoptosis by transcriptionally repressing p14ARF/p19ARF via a variant T-element [8, 24–26]. This has led to the speculation that TBX3 may bind this degenerate T-element with low affinity and that it may impact on the above processes by targeting the p19ARF-Mdm2-p53-p21^WAF^ pathway at multiple points for example by repressing p19ARF, p53 and p21^WAF^ (hereafter referred to as p21) and/or activating Mdm2 [27]. Indeed, there is evidence to suggest that TBX3 may also repress p21 transcriptionally [28] and we and others have shown an inverse correlation between the ability of TBX3 to promote proliferation of chondrosarcoma, mammary epithelial and hepatic progenitor cells and p21 levels [13, 29, 30]. However, the mechanism by which TBX3 regulates p21 has yet to be elucidated and whether this regulation is biological relevant still needs to be confirmed.

Here we show, using in vitro and in vivo assays, that p21 is a direct target of TBX3. Importantly, we provide a full characterisation of the mechanism by which TBX3 represses p21 and we demonstrate that this is required for TBX3′s pro-proliferative role in chondrosarcomas.

## Results

### TBX3 is a direct transcriptional repressor of p21 gene expression

**Fig. 1:**
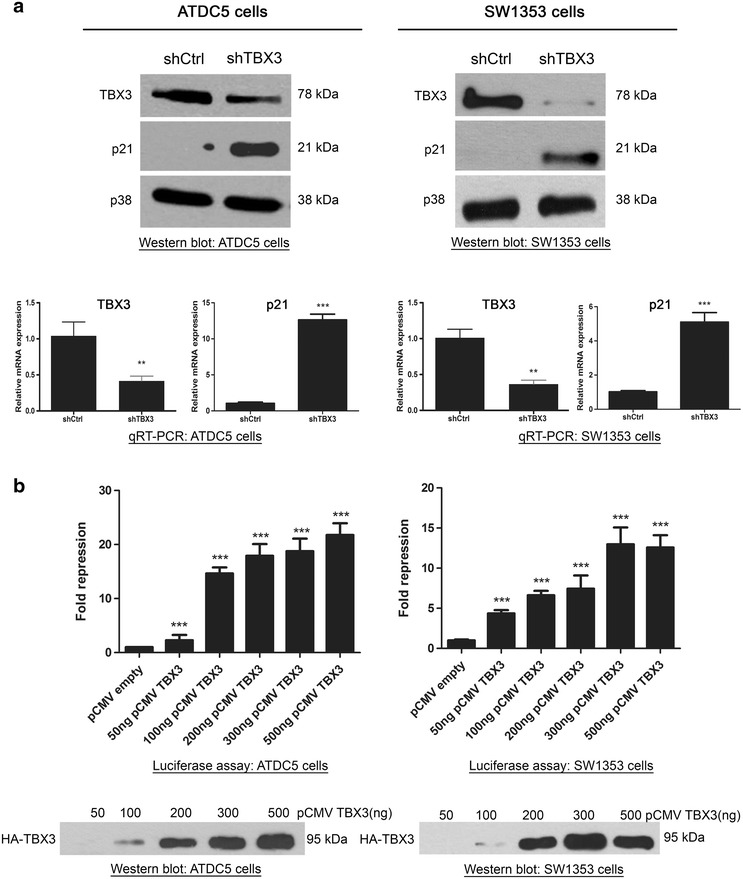
TBX3 represses p21 promoter activity. **a** Total protein and RNA were extracted from ATDC5 and SW1353 control and TBX3 knock down cells. *Upper panels* lysates were subjected to western blot analysis with antibodies specific to TBX3 and p21. p38 was used as a loading control. *Lower panels* quantitative real-time PCR was performed on reverse transcribed RNA using primers specific to TBX3 and p21 and mRNA levels were normalised against GUSB levels. **b** ATDC5 and SW1353 cells were co-transfected with the p21 promoter luciferase reporter construct and varying amounts of the TBX3 expression construct pCMV-TBX3. Total amount of plasmid DNA transfected was held constant using the corresponding empty vector, pCMV. The plasmid pRL-TK containing the Renilla luciferase reporter gene was also introduced to normalize transfection efficiency. Thirty hours following transfection cells were lysed and relative luciferase activity measured. Data were normalised against renilla values and fold repression was obtained by comparing the results to that of the empty pCMV vector transfection. *Lower panels* western blotting shows expression of HA-tagged TBX3 protein using a HA antibody. **a**, **b** The *values* in the* graphs* indicate the mean of three independent experiments ± SEM (*p < 0.05; **p < 0.01; ***p < 0.001)

**Fig. 2:**
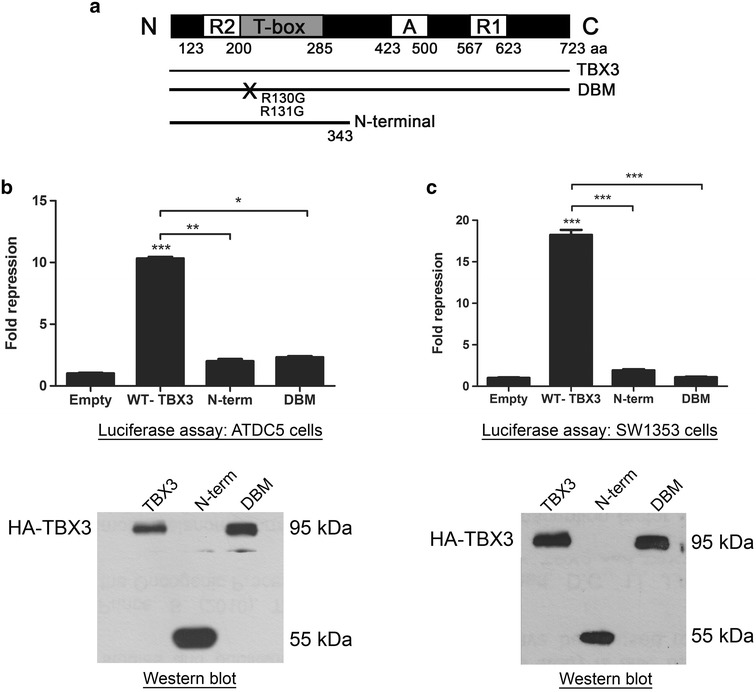
The DNA binding and repression domains of the TBX3 protein are required for the repression of p21. **a** Schematic representation of the human wild-type (WT) TBX3 protein (WT-TBX3), TBX3 N terminal (TBX3 N-term) and TBX3 DNA-binding domain mutant (DBM) expression constructs. “A” and “R” denotes activation and repression domains respectively. **b** ATDC5 and **c** SW1353 cells were co-transfected with a p21 promoter luciferase reporter construct together with pCMV empty, WT TBX3, TBX3 DBM or TBX3 N-term expression constructs. The plasmid pRL-TK containing the Renilla luciferase reporter gene was also introduced to normalize transfection efficiency. Fold repression was obtained by comparing the results to that of the empty pCMV vector transfection. **b**, **c** The *values* indicate the mean of three independent experiments ± SEM (*p < 0.05; **p < 0.01, ***p < 0.001). *Lower panels* western blotting shows expression of HA-tagged WT TBX3, TBX3 N-term and TBX3 DBM proteins using a HA antibody

To explore if p21 is transcriptionally repressed by TBX3 we first compared the protein and mRNA levels of p21 in chondrosarcoma cells in which TBX3 was stably knocked down (shTBX3) to that of control cells (shCtrl). We used the ATDC5 and SW1353 chondrosarcoma cell lines because we recently reported that TBX3 promotes their proliferation and we showed that this was accompanied by a decrease in p21 protein levels [13]. Indeed, western blot and qRT-PCR analyses show that p21 mRNA levels are dramatically upregulated in shTBX3 cells compared to control cells (Fig. 1a). We next tested the effect of TBX3 expression on a p21 promoter driving a luciferase reporter and confirm that TBX3 can repress the p21 promoter in a dose-dependent manner (Fig. 1b). Furthermore, a TBX3 mutant which has a disrupted DNA-binding domain and a N-terminal TBX3 protein lacking the R1 dominant repression domain (Fig. 2a) had a significantly reduced ability to repress the p21 promoter (Fig. 2b). Comparable protein expression was observed for all three constructs tested in the luciferase assays. This data suggest that TBX3 regulates p21 by directly binding to it and that the dominant repression domain R1 is important for p21 repression.

### TBX3 binds the p21 promoter in vitro and in vivo

**Fig. 3:**
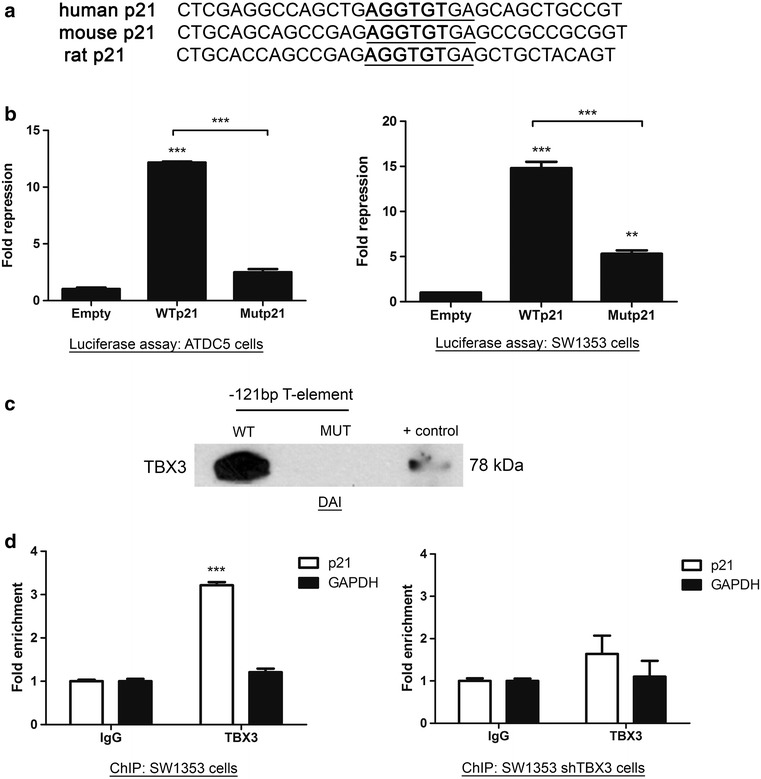
The T-element at -121 bp of the p21 promoter mediates repression by TBX3 and TBX3 binds this region in vitro and in vivo. **a** Alignment showing conservation of the T-element residue at -121 bp (T-121 bp) of the p21 promoter across a number of different species. **b** ATDC5 and SW1353 cells were co-transfected with pGL3 basic empty, WTp21 or Mutp21 luciferase reporter constructs together with pCMV empty or WT TBX3. The plasmid pRL-TK containing the Renilla luciferase reporter gene was also introduced to normalize transfection efficiency. Fold repression was obtained by comparing the results to that of the empty pCMV vector transfection. The values indicate the mean of three independent experiments ± SEM (*p < 0.05; **p < 0.01, ***p < 0.001). **c** DNA-affinity immunoblot assay. Lysates from SW1353 cells were incubated with a biotinylated probe matching the sequence of the wild-type or mutated T-element at position -121 bp of the p21 promoter. A pull-down was performed using streptavidin magnetic beads and the lysates run on an 8 % SDS-PAGE gel and analysed by western blotting using an antibody to TBX3. **d** SW1353 control and shTBX3 lysates were used in a ChIP assay performed with antibodies against TBX3 or IgG (negative control). Immunoprecipitated DNA was assayed by qRT-PCR with primers against the p21 promoter or GAPDH (negative control). The *values* indicate the mean of two independent experiments each performed in triplicate ± SEM (***p < 0.001)

A conserved consensus T-element at position -121 bp close to the initiator of the p21 promoter, from here on referred to as T-121 bp (Fig. 3a), was previously shown to be important for the binding and regulation of p21 by TBX2, the highly homologous TBX3 partner [27]. Furthermore, results from in vitro assays have also previously implicated it as a TBX3 target site [28, 31]. To confirm that this is the case, cells were co-transfected with a TBX3 expression construct and either a wild-type T-121 bp (WTp21) or mutated T-121 bp (Mut21) promoter-luciferase reporter (as previously described in [27]) after which luciferase activity was measured. Indeed, we demonstrate that T-121 bp mediates repression by TBX3 because mutating it (GTGTGA -> CTCTGA) led to almost complete abrogation of repression in ATDC5 cells and a 65 % loss in repression in SW1353 cells (Fig. 3b). Together these results indicate that T-121 bp plays a key role in mediating p21 repression by TBX3 and that in the human SW1353 cells other sites may also be important. To investigate whether TBX3 binds T-121 bp a DNA affinity immunoprecipitation (DAI) assay was performed with nuclear extracts from SW1353 cells incubated with biotinylated DNA probes containing either a wild-type T-121 bp (WT) or mutated T-121 bp (MUT). Protein-bound biotinylated DNA was isolated and analysed by western blotting using an anti-TBX3 antibody and the results show that TBX3 only bound to probes carrying a WT T-121 bp (Fig. 3c).

In order to validate this result in vivo, chromatin immunoprecipitation assays were performed. TBX3-bound DNA was immunoprecipitated from SW1353 control and SW1353 shTBX3 cells and the DNA was subjected to qRT-PCR with primers spanning a region of the p21 promoter containing the T-121 bp. The results show that in the control cells, there was an approximately 3.2 fold enrichment of TBX3 occupancy on the p21 promoter but not on the GAPDH control (Fig. 3d). As expected, this occupancy was significantly reduced in shTBX3 cells. These data suggest that, in vivo, TBX3 binds a region of the p21 promoter containing the T-121 bp.

### Pseudo-phosphorylation of TBX3 at Serine-Proline 190 (SP190) regulates its ability to repress p21

**Fig. 4:**
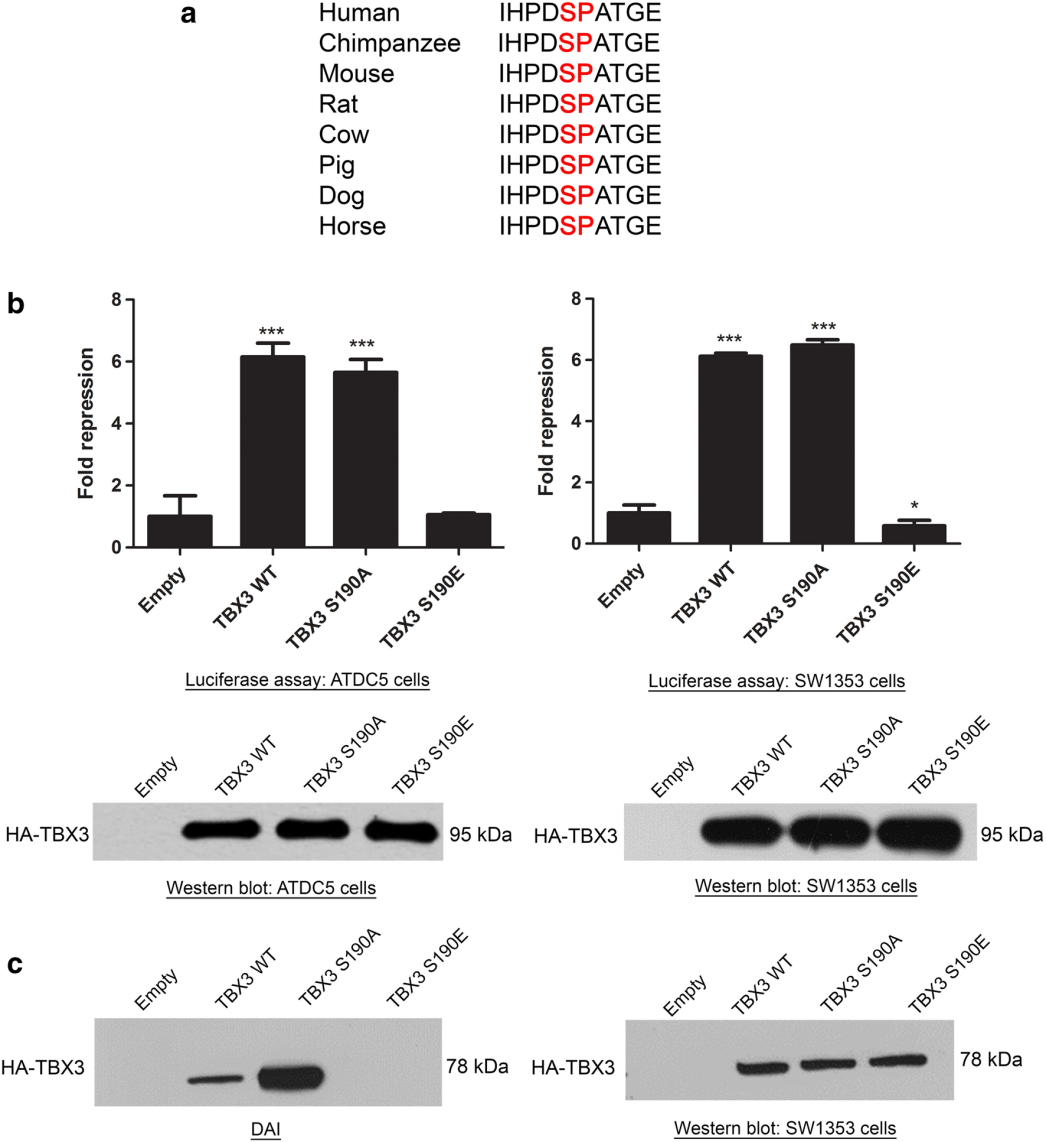
Phosphorylation of TBX3 at SP190 can affect its ability to repress p21. **a** Alignment showing conservation of the S190 residue (indicated as SP in *red*) of the TBX3 protein across a number of different species. **b** ATDC5 and SW1353 cells were co-transfected with a p21 promoter luciferase reporter construct together with pCMV empty, TBX3 WT, TBX3 S190A or TBX3 S190E expression constructs. The plasmid pRL-TK containing the Renilla luciferase reporter gene was also introduced to normalize transfection efficiency. Fold repression was obtained by comparing the results to that of the empty pCMV vector transfection. For luciferase analyses, the values indicate the mean of three independent experiments ± SEM (*p < 0.05 and ***p < 0.001). *Lower panels* western blots of transfected HA tagged-TBX3 proteins using an antibody to HA. **c** DNA-affinity immunoblot assay. *Left panel* lysates from SW1353 cells transfected with empty vector, TBX3WT, TBX3 S190A or TBX3 S190E constructs were incubated with a biotinylated probe matching the T-element at position -121 bp of the p21 promoter. A pull-down was performed using streptavidin magnetic beads and the lysates run on an 8 % SDS-PAGE gel and analysed by western blotting with an antibody to HA. *Right panel* western blotting to confirm the equal expression of the HA- tagged WT TBX3, TBX3 S190A and TBX3 S190E constructs using an antibody to HA

To further characterise the mechanism by which TBX3 represses p21, the possibility was considered that phosphorylation of TBX3 may regulate its ability to bind its target genes. This approach was based on evidence in our laboratory that suggests that TBX3 protein levels and function are regulated by phosphorylation [17]. Of particular interest was a highly conserved serine proline motif at residue 190 (SP190) because it resides within the DNA-binding domain of the TBX3 protein (Fig. 4a). To test whether phosphorylation at SP190 regulates TBX3′s ability to repress p21, site-directed mutagenesis was performed in which this serine was either mutated to an alanine (A), to abolish phosphorylation, or a glutamic acid (E), to mimic phosphorylation. The effect of these mutations was assessed using luciferase assays where chondrosarcoma cells were co-transfected with the p21 promoter luciferase reporter construct and the pCMV empty vector, WT, S190A or S190E TBX3 expression constructs. Figure 4b shows that while the WT TBX3 and TBX3 S190A proteins repressed the p21 promoter, the pseudophosphorylated mutant (TBX3 S190E) had severely reduced repressive ability. To exclude the possibility that the results obtained may be due to different levels of TBX3 expression for the different constructs, lysates used for the luciferase assays were also subjected to western blotting. Results show that equal protein expression was observed for each of the constructs (lower panel of Fig. 4b). These results suggest that phosphorylation of SP190 does indeed play a role in regulating the transcriptional activity of TBX3 on the p21 promoter. The possibility that phosphorylation at SP190 may be interfering with the ability of TBX3 to bind to the p21 promoter was further investigated using a DAI assay in which lysates from SW1353 cells transfected with the pCMV empty, WT, S190A or S190E TBX3 constructs were incubated with biotinylated p21 T-121 bp probes. Results show that whereas WT and S190A TBX3 proteins bind the T-121 bp, the TBX3 S190E protein was unable to bind this site (Fig. 4c, left panel). To exclude the possibility that the results obtained may be due to different levels of TBX3 expression for the different constructs, lysates used for the DAI assays were also subjected to western blotting. Results show that equal protein expression was observed for each of the constructs (Fig. 4c, right panel). Together the results suggest that phosphorylation of TBX3 at SP190 abolishes its binding and consequently repression of the p21 promoter.

### Down-regulation of p21 by TBX3 mediates its pro-proliferative effects in chondrosarcoma cells

**Fig. 5:**
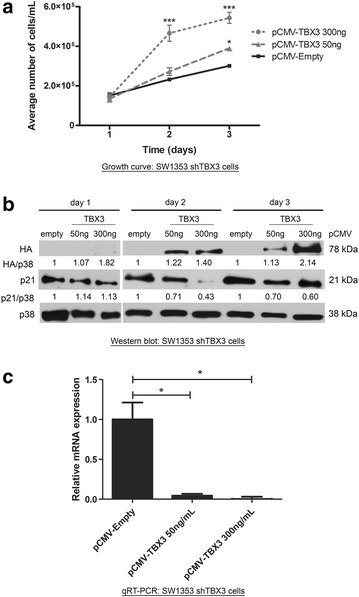
TBX3 promotes chondrosarcoma cell proliferation in a dose dependent manner which correlates inversely with p21 protein and mRNA levels. **a** SW1353 shTBX3 cells were transiently transfected with either 50 ng or 300 ng pCMV-TBX3 and growth curve assays were performed over a 3 day period. **b** Western blotting and **c** qRT-PCR show a correlation between cell proliferation and the expression of transfected TBX3 and p21 protein and mRNA levels in SW1353 shTBX3 cells. *Error bars* indicate standard deviation (*p < 0.05 and ***p < 0.001)

**Fig. 6:**
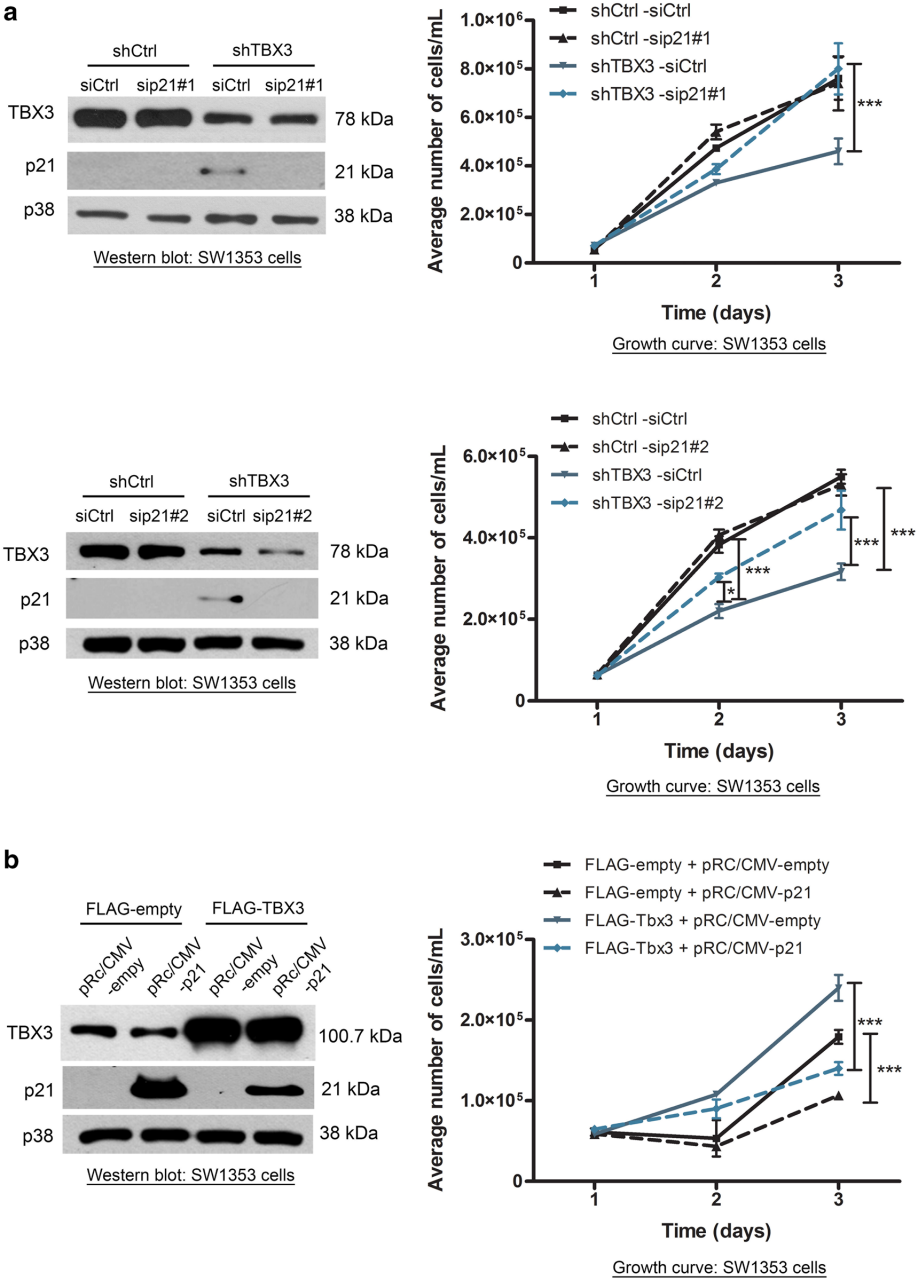
The repression of p21 is required for TBX3 induced cell proliferation. **a** SW1353 cells were transiently transfected with either 25 nM sip21 or siControl for 12 h. Growth curve assays were performed over a 3 day period (*upper panel* sip21#1, *lower panel* sip21#2.). **b** SW1353 cells were transiently transfected with either pRc/CMV-Empty (control) or pRc/CMV-p21 (p21). Growth curve assays were performed over a 3 day period (*right panels*). (**a**, **b**) Two way ANOVA test was performed to calculate statistical significance (***p < 0.001). *Left panels* western blotting to confirm the expression of the indicated proteins using antibodies to TBX3, p21 and p38 (loading control)

We recently reported that knocking down TBX3 inhibited proliferation of chondrosarcoma cells and resulted in the re-expression of key cell cycle regulators including p21 [13]. This suggested that TBX3 may promote the proliferation of chondrosarcoma cells by, in part, repressing p21. Indeed, Fig. 5 shows that there is a direct correlation between transfected TBX3 levels in shTBX3 cells and cell proliferation and that this correlated inversely with p21 protein and mRNA levels. Furthermore, growth curve assays show that while knocking down TBX3 significantly reduced cell proliferation, dual silencing of TBX3 and p21 was sufficient to partially rescue this phenotype (Fig. 6a). Consistent with these findings, ectopic expression of p21 could inhibit proliferation of SW1353 cells stably over-expressing TBX3 (FLAG-TBX3) (Fig. 6b). Together these data suggest that the repression of p21 by TBX3 is a key downstream event in mediating the ability of TBX3 to promote proliferation of chondrosarcoma cells.

### Pseudo-phosphorylation of TBX3 at SP190 reduces its ability to promote cell proliferation

**Fig. 7:**
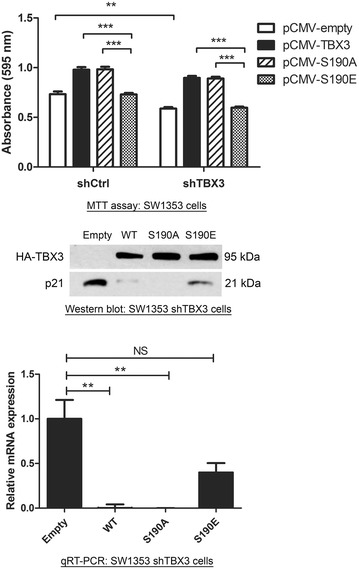
Phosphorylation of TBX3 at SP190 reduces its ability to promote cell proliferation in a p21-dependent manner. *Upper panel* SW1353 shCtrl and shTBX3 cells were co-transfected with pCMV empty, WT TBX3 (pCMV-TBX3), TBX3 S190A (pCMV-S190A) or TBX3 S190E (pCMV-S190E) expression constructs. Twenty four hours following transfection cells were replated at 4000 cells/well in 96 well plates and allowed to adhere for 24 h. Cells were subjected to MTT and metabolization measured at OD 595 nm. The values indicate the mean of three independent experiments ± SEM (**p < 0.01 and ***p < 0.001). *Lower panel* western blotting and qRT-PCR showing a correlation between the expression of WT TBX3, TBX3 S190A and TBX3 S190E and p21 protein and mRNA levels in SW1353 shTBX3 cells. *Error bars* indicate standard deviation (**p < 0.01) and NS indicates no statistical significance

Based on the observation that the TBX3 S190E mutant had a reduced ability to bind and repress the p21 promoter, we hypothesised that this pseudo-phosphorylated TBX3 may not be able to promote chondrosarcoma cell proliferation. To test this, SW1353 shCtrl and shTBX3 cells were transfected with pCMV empty, WT, S190A or S190E TBX3 expression constructs and short term cell proliferation assays were performed. Indeed, results show that unlike WT and S190A TBX3 proteins, TBX3 S190E had no effect on cell proliferation (Fig. 7). Importantly, western blotting and qRT-PCR analyses show that endogenous p21 is repressed by WT and S190A TBX3 proteins but not by the S190E TBX3. Collectively these results suggest that the phosphorylation of TBX3 at SP190 regulates its ability to repress p21 and that this is an important determinant of its ability to promote proliferation of chondrosarcoma cells.

## Discussion

Despite a growing list of reports implicating TBX3 in development and oncogenesis, little is known about the molecular basis for its role in these processes because there is a paucity of information regarding its target genes. Of relevance to this study is the evidence suggesting that during embryonic development and cancer, TBX3 impacts on cell proliferation by repressing key cell cycle regulators [26, 28, 29, 32–34]. For example, an intact p19ARF-Mdm2-p53-p21 pathway is important for protecting cells from immortalisation and results from a previous study revealed that depleting TBX3 in chondrosarcoma cells led to an increase in p19ARF, p53 and p21 protein levels which was accompanied by a decrease in cell proliferation [13]. This raised the possibility that TBX3 may promote cell proliferation by disabling this pathway at multiple points for example by repressing p19ARF, p53 and p21 and/or activating Mdm2. Indeed, TBX3 has been reported to directly repress p14ARF/p19ARF and our observation that p21 levels increase when TBX3 is depleted in the mouse chondrosarcoma ATDC5 cells, which do not express p53 [13], implies that TBX3 must also repress p21 independently of p19ARF-p53. While this is consistent with other reports suggesting that p21 may be a direct TBX3 target gene [14, 35], the mechanism by which TBX3 regulates p21 and the biological context in which this regulation occurs has yet to be elucidated. The current study shows that p21 mRNA and protein levels increased when TBX3 was knocked down in chondrosarcoma cells and provides compelling in vitro and in vivo data to show that TBX3 directly represses p21 which is important for TBX3-induced cell proliferation.

This study provides a full characterisation of the repression of p21 by TBX3 in chondrosarcoma cells. Consistent with p21 being a direct TBX3 target gene, the TBX3 DNA binding domain (T-box) was shown to be required for this repression. Interestingly, Pflugfelder and Fischer also recently reported that two ulnar mammary syndrome associated TBX3 DNA binding domain mutations (H187Y and R130S/G129A) could both abrogate its ability to repress a p21 promoter driving a luciferase reporter [36]. Furthermore, we show that a highly conserved T-element at -121 bp mediates, in part, the repression of p21 by TBX3. This site was previously shown to be required for the repression of p21 by TBX2 but we have been unable to detect TBX2 protein in ATDC5 or SW1353 cells and therefore it is unlikely to be regulating p21 in these cells [27]. It would therefore be interesting to determine whether the highly homologous TBX2 and TBX3 have redundant functions in regulating the p21 promoter at this site in cancers where they are both expressed or whether the availability of cofactors may determine which one of the two regulates p21. The repression domain (RD1) in the C-terminus of the TBX3 protein was also shown to be required for the repression of p21. This is consistent with reports that have indicated that of the two repression domains in the TBX3 protein, RD1 is the dominant one [31]. Indeed, RD1 was also recently reported to be required for the repression of TBX2 by TBX3 and it has previously been shown to be essential for the ability of TBX3 to inhibit apoptosis and immortalise primary embryonic fibroblasts [31, 35, 37]. Furthermore, the majority of ulnar mammary syndrome-associated TBX3 C-terminal mutants that exhibit loss of function have been reported to lack the dominant repression domain [14].

Using rescue assays in TBX3 knockdown and overexpression chondrosarcoma cell culture models, the repression of p21 by TBX3 was shown to be physiologically relevant because it was required for TBX3-induced cell proliferation. Importantly, a highly conserved serine-proline motif at amino acid 190 in the TBX3 DNA-binding domain (SP190) was shown to regulate its ability to bind and transcriptionally repress p21 and consequently, its ability to promote proliferation. Indeed, when the serine residue at this site was mutated to glutamic acid (E) to mimic phosphorylation, the ability of TBX3 to repress the p21 promoter was abrogated and this mutant could also not promote proliferation. SP motifs are consensus sites for many kinases including the MAP kinases [38] and CDKs [39] and this data suggest that post-translational mechanisms such as phosphorylation may be important to regulate TBX3 function. Indeed, there is evidence that phosphorylation of TBX3 regulates its ability to transcriptionally repress its target genes. For example, phosphorylation of TBX3 at SP692 and SP720 by the p38 mitogen-activated protein (MAP) kinase and AKT3 respectively can enhance its ability to transcriptionally repress its well-known target, E-cadherin [17, 40]. The kinase responsible for phosphorylating TBX3 at SP190 was not identified in this study however, we are currently investigating the possibility that it may be ERK which, like TBX3, can act as either an oncoprotein or a tumour suppressor [13]. Furthermore, ERK has been shown to negatively regulate cell proliferation in human hepatoma cells [41, 42]. It is therefore possible that phosphorylation of TBX3 by ERK may provide a mechanism by which ERK can inhibit proliferation and future experiments should investigate this possibility because it has implications for our understanding of TBX3′s oncogenic role.

It is important to note that p21 expression and activity are tightly regulated both transcriptionally and post-transcriptionally and we therefore cannot exclude the possibility that, in addition to TBX3, p21 may be subject to regulation by other factors in our study. Indeed, in response to stimuli such as DNA damage, oxidative stress, cytokines and mitogens, p21 can be transcriptionally regulated by p53, Sp1, Sp3, p63, AP4, Klf, and c-Myc and post-translationally by E3 ubiquitin ligase complexes and phosphorylation by AKT1 [43, 44]. Furthermore, in chondrosarcomas as well as in other cancers, TBX3 has been implicated in several oncogenic processes including promoting cell proliferation, tumour formation, migration and invasion which suggest that it must be regulating several other target genes. Indeed, it is possible that in addition to p21, TBX3 may promote cell proliferation by repressing other negative regulators of the cell cycle such as PTEN, p14ARF/p19ARF and p53.

## Conclusions

TBX3 is essential in development and has emerged as an important player in the genesis of several cancers where it directly contributes to proliferation, tumour formation, migration and invasion. However, little is known about the molecular basis for its roles in these processes because its full repertoire of target genes has not been identified. Here we show that TBX3 promotes proliferation by directly repressing the CDKI p21. This repression is shown to involve the TBX3 DNA binding domain and is mediated by a T-element at -121 bp of the p21 promoter. The significance of our findings may have implications for the design of anti-cancer drugs to treat TBX3-driven cancers.

## Methods

### Plasmid constructs

The wild-type *p21* luciferase reporter construct (WTp21) contains a 2.4 kb fragment of the human p21 promoter inserted upstream of a 2.6 kb luciferase (LUC) reporter gene [45]. This construct was kindly provided by Professor Chris Marshall of the Institute of Cancer Research, UK. The mutant *p21* luciferase reporter construct (Mutp21) contains a disrupted T-element in the initiator of the *p21* promoter generated by site-directed mutagenesis as previously described [30]. The p21-pRc/CMV plasmid, which was prepared in the laboratory of Prof William Kaelin, was obtained from Addgene (Addgene plasmid 20814) [18, 46]. The human pCMV-TBX3-HA expression construct was kindly provided by Dr Christine Campbell of the Cleveland Clinic Foundation, USA. The TBX3 DNA-binding mutant (DBM) was generated by site-directed mutagenesis using the following primer 5′ CATTACCAAGTCGGGAgGGgGAATGTTTCCTCCATTTAAAG 3′. Using the pCMV-TBX3-HA expression construct, the TBX3 C-Terminal region was removed by cutting with BglII (at position 1121 bp) and SpeI (50 bp after stop translation site) thus generating the TBX3 N-terminal construct. The TBX3 SP190A and E mutant constructs were generated by site-directed mutagenesis using the following primers: SP190A: 5′CATTCACCCGGACgcgCCCGCTACTGGGGAACAGTGGATG3′, SP190E: 5′CATTCACCCGGACgagCCGGCTACTGGGGAACAGTGGATG3′.

### Cell lines and culture condition

SW1353 chondrosarcoma cells (American Type Culture Collection) were cultured in Dulbecco’s modified Eagle’s medium (DMEM) (Highveld Biological), supplemented with 10 % fetal bovine serum, 100 U/ml penicillin and 100 µg/ml streptomycin. ATDC5 chondrosarcoma cells were maintained in Dulbecco’s modified Eagle medium: nutrient mixture F-12 (DMEM/F12) (1:1) supplemented with 5 % FBS, 100 U/ml penicillin, 100 µg/ml streptomycin, 10 μg/ml human transferrin (T Sigma Alderich, USA), and 3 × 10^−8^ M sodium selenite (Sigma Alderich, USA). All cells were maintained in a 37 °C incubator (95.0 % air/5 % CO2, 65 % humidity).

### Generation of stable TBX3 knock down cell lines

Stable knockdown of TBX3 in SW1353 and ATDC5 cell lines was achieved with the following siRNA sequence cloned into the pSuper.neo/GFP (Oligoengine) shRNA expression vector as previously described [11, 13]. Cells were transfected with the pSuper.neo/GFP expression vector containing sequences targeted to TBX3, or the nonspecific control using FugeneHD (Roche Diagnostics, Basel, Switzerland). Stable transfectants were selected by the addition of 400 μg/ml (ATDC5 cells) or 800 μg/ml (SW1353 cells) of G418 (Invitrogen) to the growth medium.

### Generation of stable TBX3 overexpression cell lines

Tbx3 overexpressing cell lines were generated by stably transfecting SW1353 cells with a FLAG-tagged pCMV-empty vector, pCMV-Tbx3 (kindly provided by Professor Colin Goding at the Ludwig Institute of Cancer Research, UK) using FuGENE^®^HD (Roche Molecular Biochemicals, Mannheim, Germany) according to the manufacturer’s instructions. Stable transfectants were selected for with 800 μg/ml G418 antibiotic (Promega, USA). Effective overexpression of Tbx3 was assessed by western blot analysis.

### Transfections and luciferase assays

SW1353 or ATDC5 cells were plated at 7 × 10^4^ cells per well in a 12 well plate. One day later cells were transiently transfected using XtremeGENE HP DNA transfection reagent (Roche), according to the manufacturer’s instructions. 200 ng of the relevant *p21* reporter plasmid and varying amounts of the TBX3 expression construct or empty vector were transfected. 10 ng of the RL-CMV vector (Promega), which contains the cytomegalovirus promoter driving the expression of a renilla reporter, was used as an internal control for transfection efficiency. The Dual-Luciferase Reported Assay System (Promega, USA) and a Luminoskan Ascent luminometer (Thermo Labsystems) were used to determine relative luciferase and renilla activity.

### Small interferring RNA (siRNA) sequences and transfections

Transient knockdown of p21 expression was achieved using ON-TARGETplus SMARTpool Human CDKN1A siRNA (sip21#1) (Thermo Scientific) and sip21#2 (SI00299810) from Qiagen. shTBX3, shCtrl, FLAG-TBX3 and FLAG-Empty cells were transfected siRNAs to p21 or control ((non-silencing) siRNA (1027310; Qiagen) using HiPerFect (Qiagen) according to the manufacturer’s instructions.

### Chromatin immunoprecipitation assay (ChIP)

90 % confluent SW1353 cells were fixed with formaldehyde, washed in PBS and collected by centrifugation. Following this the chromatin was extracted and subjected to sonication to obtain DNA fragments of between 300 and 500 bp. The protein-bound DNA was then immunoprecipitated with A/G PLUS Agarose beads (Santa Cruz Biotechnology Inc) and an antibody against TBX3 (8ug, sc-17871, Santa Cruz Biotechnology Inc.) or IgG (8ug, negative control, Santa Cruz Biotechnology) was added overnight. The beads were then washed and the cross-links reversed by heating at 65 °C. The DNA to which TBX3 bound was purified using the PCR purification kit (Qiagen, USA) according to the manufacturer’s instructions. qRT-PCR analysis was performed using primers spanning the T-element in the initiator of the *p21* promoter (Forward-5′-GGGGCGGTTGTATATCAGGG-3′, Reverse-5′-TCTCACCTCCTCTGAGTGCC-3′). Crossing values (Ct) of TBX3 and IgG DNA were adjusted by normalising against the Ct value of 1 % of input DNA and the ΔΔCt method was used to determine fold enrichment. The equation was used as follows: 2^−(∆Ct1−∆Ct2)^ (∆Ct1 = TBX3, ∆Ct2 = IgG).

### Western blot analyses

Cells were harvested and protein prepared as described previously [45]. Primary antibodies used were as follows: rabbit polyclonal anti-TBX3 (Zymed 42-4800 or Abcam 99302) and polyclonal rabbit anti-p38 (Sigma-Aldrich, M0800), monoclonal mouse anti-HA (Sigma-Aldrich, H9658), and polyclonal rabbit anti-p21 (Santa Cruz Biotechnology Inc., C-19). Signal was detected using peroxidase-conjugated goat anti-mouse or anti-rabbit antibodies (1:5000) and visualised by enhanced chemiluminescence (ECL) (Pierce, USA).

### Quantitative real-time polymerase chain reaction (qRT-PCR)

RNA was extracted from cells using the high pure RNA isolation kit (Roche, Germany) according to the manufacturer’s instructions and reverse transcription performed using 1ug of RNA and the InProm-II™ reverse transcription system (Promega A3800, USA) according to the manufacturer’s instructions. The reactions were carried out using 2 μl of cDNA, Power SYBR^®^ Green PCR Master Mix (Applied Biosystems, UK) and primers to amplify the human TBX3 and p21 genes (Qiagen, USA). qRT-PCR was performed using the StepOnePlus™ PCR system (Applied Biosystems) using the following parameters: denaturation for 15 min at 95 °C and combined annealing and extension for 35 cycles at 60 °C for 1 min. Samples were prepared in triplicate and non-template controls were run to detect contamination or non-specific amplification. The 2^−ΔΔCt^ method employed to analyse results and relative mRNA expression levels of TBX3 and p21 were normalised to mRNA levels of glucuronidase-beta (GUSB).

### DNA affinity immunoblot (DAI)

Oligonucleotide probes p21WT (5′-CTCGAGGCCAGCTGAGGTGTGAGCAGCTGCCG-3′) and p21MUT (5′-CTCGAGGCCAGCTGAGGCTCGAGCAGCTGCCG-3′) were annealed with their complementary strands. SW1353 were harvested and nuclear extracts isolated as described previously [18]. Alternatively cells were transfected with pCMV empty vector, WT TBX3, TBX3 SP190A or TBX3 SP190E constructs and whole cell lysate harvested. 20 µg of this protein was analysed by western blotting to confirm that the constructs were expressed at equal levels in the cell before continuing. For the DAI, biotinylated p21 probe was immobilized on Dynabeads Streptavidin (Dynal Invitrogen) according to the manufacturer’s instructions. For each reaction, 40 μg protein extract was incubated with 1 μg DNA probe in binding buffer (20 mM Tris–HCL;pH7.6, 50 mM MaCl, 1 mM MgCl_2_, 0.2 mM EDTA, 5 % glycerol, 0.5 mM DTT and 2 µg poly dI/dC) in a final volume of 200 µl. The protein and probe bound beads were pulled down with a magnet, extensively washed with binding buffer and then boiled in 25 μl of 2 × SDS-PAGE sample buffer before SDS-polyacrylamide gel electrophoresis followed by immunoblotting with an antibody to HA.

### Growth curve and MTT assays

Short-term growth of cells was compared to that of controls over a 3-day period, as described previously [47]. 3 × 10^4^ cells were seeded in triplicate in 24-well plates on day 0 and treated with siRNA for 2–3 days. Alternatively cell proliferation was assessed using the MTT cell proliferation assay kit. At the appropriate times, 10 μl MTT reagent was added to each well to a final concentration of 0.5 mg/ml and cells were incubated at 37 °C in 9 % CO^2^ for 4 h. To each well, 100 μl of solubilization solution was added and the cells incubated overnight, after which the absorbance measured at 595 nm. Cell growth graphs were constructed of the cell viability as absorbance measured for each transfection treatment.

### Statistical analyses

Statistical significance was determined using the student *T* test (Excel, Windows XP). Significance was accepted at p < 0.05.
